# Supplementary material for: Compensatory mutations reducing the fitness cost of plasmid carriage occur in plant rhizosphere communities
Source: FEMS Microbiol Ecol. 2023 Mar 23;99(4):fiad027. doi: 10.1093/femsec/fiad027 (PMC10062694; doi:10.1093/femsec/fiad027)
Supplement: fiad027_Supplemental_Files [file fiad027_supplemental_files.zip › Supplementary_figure_legends.docx]

**Figure S1 | The proportion of transconjugants in initially plasmid free SBW25 competitor population after 14 days post infection.** Plasmid carrying donor strains were either the wild type SBW25, SBW25𝚫gacS or SBW25𝚫PFLU4242. Strains carried either a wild type plasmid (red) or a rsmQ knockout plasmid (pink). Boxplots show mean and interquartile range with replicate values shown as points in black (n=6).

**Figure S2 | Genome plots for Gac-negative phenotype SBW25 evolved clones sampled over time.** Concentric rings represent the chromosome of individual sequenced clones colour-coded by treatment, SBW25(pQBR103) (red) and SBW25(pQBR103𝚫*rsmQ)* (pink). The sampling day when the clone was picked is denoted by shading. Black dots denote the location of mutations with gene targets shown on the outer labels.
