# Supplementary material for: Compensatory mutations reducing the fitness cost of plasmid carriage occur in plant rhizosphere communities
Source: FEMS Microbiol Ecol. 2023 Mar 23;99(4):fiad027. doi: 10.1093/femsec/fiad027 (PMC10062694; doi:10.1093/femsec/fiad027)
Supplement: fiad027_Supplemental_Files [file fiad027_supplemental_files.zip › Tables_S1_S2.docx]

| **Treatment** | **Replicate population** | **Gene name** | **Position** | **Gene position** | **Gene product** | **Mutation type** |
| --- | --- | --- | --- | --- | --- | --- |
| Plasmid free | 1 | - |  |  |  |  |
|  | 2 | PFLU0325/hisB (PFLU0327) | 360368 | intergenic (+30/-144) | Hypothetical protein/imidazoleglycerol-phosphate dehydratase | Large deletion |
|  | 3 | PFLU2165/PFLU2167 | 2348042 | intergenic (+476/-74) | Hypothetical protein/putative lipoprotein | Large deletion |
|  | 4 | - |  |  |  |  |
|  | 5 | - |  |  |  |  |
|  | 6 | - |  |  |  |  |
|  | 7 | - |  |  |  |  |
|  | 8 | - |  |  |  |  |
| Plasmid + | 1 | - |  |  |  |  |
| pQBR103 | 2 | coda (PFLU2109) | 2290258 | 1028 | Cytosine deaminase | SNP nonsynonymous |
|  |  | PFLU2533 | 2746672 | 794 | Putative methyl-accepting chemotaxis protein | SNP nonsynonymous |
|  | 3 | - |  |  |  |  |
|  | 4 | - |  |  |  |  |
|  | 5 | - |  |  |  |  |
|  | 6 | gacA (PFLU2189) | 2372785 | 104 | Two-component system response regulator | SNP nonsynonymous |
|  | 7 | - |  |  |  |  |
|  | 8 | PFLU0325/hisB (PFLU0327) | 360368 | intergenic (+30/-144) | Hypothetical protein/imidazoleglycerol-phosphate dehydratase | Large deletion |
| Plasmid + | 1 | - |  |  |  |  |
| pQBR103*delta*rsm | 2 | - |  |  |  |  |
|  | 3 | - |  |  |  |  |
|  | 4 | gacS (PFLU3777) | 4173478 | coding (959-1411/2754 nt) | Hybrid sensory histidine kinase in two-component regulatory system with UvrY | Large deletion |
|  | 5 | PFLU2452 | 2670417 | coding (1558-1564/1905 nt) | Putative asparagine biosynthesis-like protein | Small indel |
|  | 6 | - |  |  |  |  |
|  | 7 | gacS (PFLU3777) | 4173493 | 974 | Hybrid sensory histidine kinase in two-component regulatory system with UvrY | SNP nonsynonymous |
|  | 8 | - |  |  |  |  |

Supplementary table S1. Mutations appearing in clones isolated randomly from populations isolated from plants at day 28. Mutations were identified using BreSeq, comparing individual sequenced clones to the ancestral strain. Clones with no mutations listed were identical to the ancestral sequence.

| **Treatment** | **Replicate population** | **Transfer** | **Gene name** | **Position** | **Gene position** | **Gene product** | **Mutation type** |
| --- | --- | --- | --- | --- | --- | --- | --- |
| Plasmid + | 3 | 3 | gacA (PFLU2189) | 2373236 | 555 | Two-component system response regulator | SNP nonsense |
|  | 4 | 1 | gacA (PFLU2189) | 2372692 | 11 | Two-component system response regulator | SNP nonsynonymous |
|  | 4 | 2 | gacS (PFLU3777) | 4175220 | coding (2701-2709/2754 nt) | Hybrid sensory histidine kinase in two-component regulatory system with UvrY | Small indel |
|  | 4 | 4 | gacS (PFLU3777) | 4173525 | coding (1006/2754 nt) | Hybrid  sensory histidine kinase in two-component regulatory system with UvrY | Small indel |
| Plasmid + | 3 | 3 | gacS (PFLU3777) | 4172602 | coding (83/2754 nt) | Hybrid  sensory histidine kinase in two-component regulatory system with UvrY | Small indel |
| pQBR103*delta*rsm |  |  | PFLU2452 | 2670417 | coding (1558-1564/1905 nt) | Putative asparagine biosynthesis-like protein | Small indel |
|  | 4 | 0 | gacS (PFLU3777) | 4174533 | 2014 | Hybrid  sensory histidine kinase in two-component regulatory system with UvrY | SNP nonsynonymous |
|  | 4 | 2 | gacS (PFLU3777) | 4174533 | 2014 | Hybrid  sensory histidine kinase in two-component regulatory system with UvrY | SNP nonsynonymous |
|  |  |  | PFLU0483/PFLU0485 | 550359 | intergenic (+654/+121) | Putative lipopolysaccharide biosynthesis-related epimerase/dehydratase/hypothetical protein | SNP intergenic |
|  | 5 | 4 | Dxr (PFLU1276) | 1411479 | 453 | 1-deoxy-D-xylulose 5-phosphate reductoisomerase | SNP synonymous |
|  |  |  | PFLU2452 | 2670417 | coding (1558-1564/1905 nt) | Putative asparagine biosynthesis-like protein | Small indel |
|  | 6 | 4 | gacS (PFLU3777) | 4173478 | coding (959-1411/2754 nt) | Hybrid  sensory histidine kinase in two-component regulatory system with UvrY | Large deletion |

Supplementary table S2. Mutations appearing in gac-negative clones isolated during experiment. Mutations were identified using BreSeq, comparing individual sequenced clones to the ancestral strain. Clones with no mutations listed were identical to the ancestral sequence.
