# Supplementary figures and images for: Compensatory mutations reducing the fitness cost of plasmid carriage occur in plant rhizosphere communities
Source: FEMS Microbiol Ecol. 2023 Mar 23;99(4):fiad027. doi: 10.1093/femsec/fiad027 (PMC10062694; doi:10.1093/femsec/fiad027)

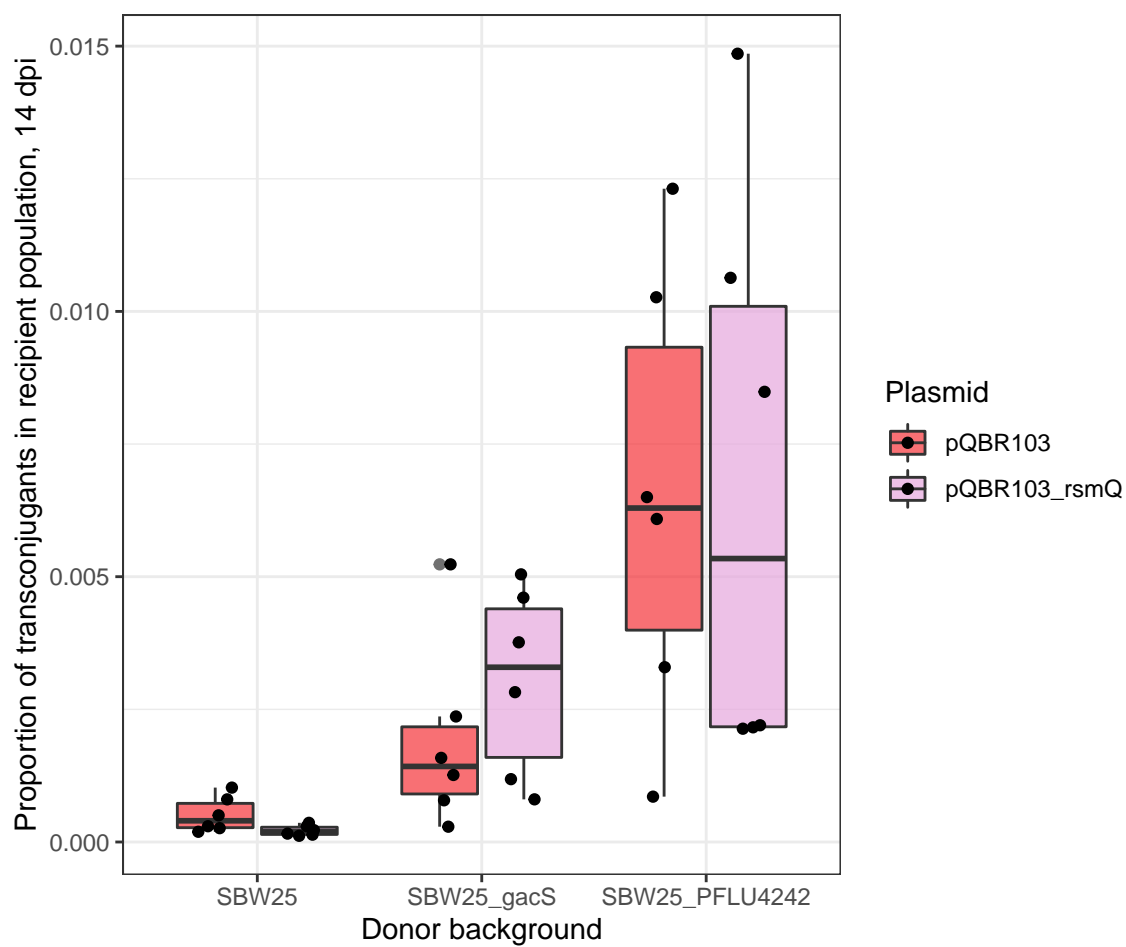

Supplement: fiad027_Supplemental_Files [file fiad027_supplemental_files.zip › Figure_S1.pdf]

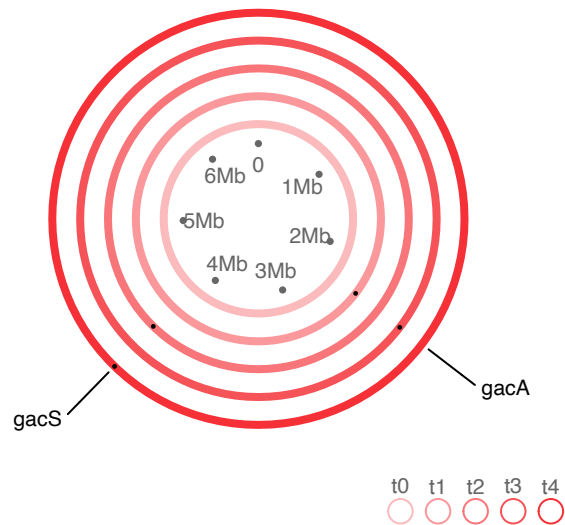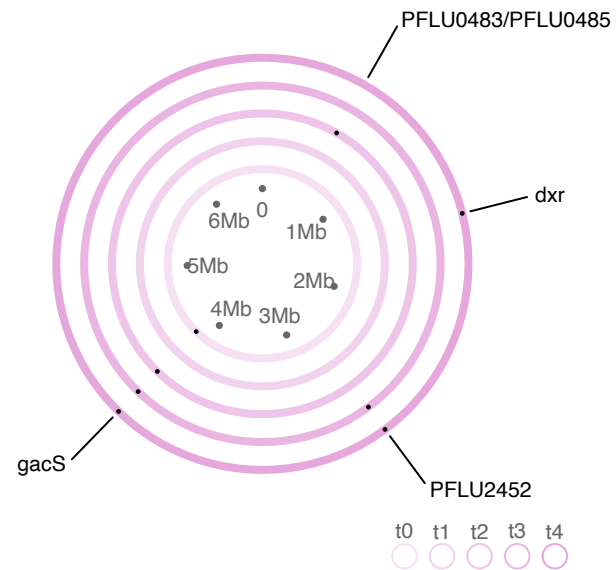

Supplement: fiad027_Supplemental_Files [file fiad027_supplemental_files.zip › Figure_S2.pdf]
